# New Experiments and a Model-Driven Approach for Interpreting Middle Stone Age Lithic Point Function Using the Edge Damage Distribution Method

**DOI:** 10.1371/journal.pone.0164088

**Published:** 2016-10-13

**Authors:** Benjamin J. Schoville, Kyle S. Brown, Jacob A. Harris, Jayne Wilkins

**Affiliations:** 1 Centre for Excellence in Palaeosciences Postdoctoral Fellow, Department of Archaeology, University of Cape Town, Cape Town, Private Bag, Rondebosch, 7701, South Africa; 2 Human Evolution Research Institute, Department of Archaeology, University of Cape Town, Cape Town, Private Bag, Rondebosch, 7701, South Africa; 3 Institute for Human Origins, School of Human Evolution and Social Change, Arizona State University, Tempe, Arizona, 85287, United States of America; Universidade do Algarve, PORTUGAL

## Abstract

The Middle Stone Age (MSA) is associated with early evidence for symbolic material culture and complex technological innovations. However, one of the most visible aspects of MSA technologies are unretouched triangular stone points that appear in the archaeological record as early as 500,000 years ago in Africa and persist throughout the MSA. How these tools were being used and discarded across a changing Pleistocene landscape can provide insight into how MSA populations prioritized technological and foraging decisions. Creating inferential links between experimental and archaeological tool use helps to establish prehistoric tool function, but is complicated by the overlaying of post-depositional damage onto behaviorally worn tools. Taphonomic damage patterning can provide insight into site formation history, but may preclude behavioral interpretations of tool function. Here, multiple experimental processes that form edge damage on unretouched lithic points from taphonomic and behavioral processes are presented. These provide experimental distributions of wear on tool edges from known processes that are then quantitatively compared to the archaeological patterning of stone point edge damage from three MSA lithic assemblages—Kathu Pan 1, Pinnacle Point Cave 13B, and Die Kelders Cave 1. By using a model-fitting approach, the results presented here provide evidence for variable MSA behavioral strategies of stone point utilization on the landscape consistent with armature tips at KP1, and cutting tools at PP13B and DK1, as well as damage contributions from post-depositional sources across assemblages. This study provides a method with which landscape-scale questions of early modern human tool-use and site-use can be addressed.

## Introduction

The human niche is broad and includes an array of plants and animals captured using many technological adaptations. Specifically, this niche includes the tools needed to dispatch, disarticulate, and distribute animal protein. Besides nutritional value, animal products are used as a commodity for prestige [1, 2], gift giving [3, 4], and other social activities [5, 6]. Technological innovations that improve the ability of foragers to efficiently acquire such resources provide fitness gains. Resource extraction is pivotal to understanding foraging economies, therefore inferring how stone tools were used provides insight into the spatial and temporal context of the fitness enhancing benefits of lithic technologies.

The earliest evidence for complex, symbolic behaviors are from Middle Stone Age (MSA) assemblages in South Africa [7]. Personal ornamentation, abstract designs, and ochre painting equipment from MSA sites suggest that these populations were symbolling and interacting with each other in similar ways as much more recent human groups [8, 9]. The technologies employed by these populations provide insight into how they structured their resource acquisition activities, which is fundamental to how they were utilizing the changing Pleistocene landscape.

One of the most visible aspects of MSA technologies are unretouched triangular stone points. MSA points are often thought of as spear tips for hunting [7, 10]. Analysis of the points from Sibudu Cave [11, 12], Blombos Cave [13], Rose Cottage Cave [14], and Kathu Pan 1 [15] have emphasized the use of points as hunting implements. A piece of stone embedded in a cervical *Pelorovis* vertebra at Klasies River from Cave 1 MSA levels supports this interpretation [10]. O’Driscoll’s [16, 17] experiments suggested embedded stone during butchery at Klasies River is unlikely, and argue that it was caused by projectile impact damage, despite its unusual position [18]. Others have noted pointed lithic forms likely served several functions in the MSA, as projectiles do ethnographically [19], and analyses by Kuman at ≠Gi and Florisbad [20], Bird et al. and Schoville at PP13B [21, 22], and Iovita in North Africa [23] indicate points were often used as cutting tools. Milo [10] presents 17 instances of embedded stone in the Klasies River faunal assemblage where butchery was inferred based on similarities with Milo’s own butchery experiments, which may imply stone embedded in faunal remains due to both armatures and butchery. Abundant, large-bodied fauna from MSA archaeological sites implicate humans as the primary accumulator [24, 25], including many difficult to acquire taxa [26]. Sites such as Pinnacle Point Cave 13B, and Florisbad, where points are argued to have not been used as armature tips still have large game [27, 28], presumably from active hunting (c.f. [29]).

Technological organization is constrained by human land-use patterns because there is a finite amount of material that can be carried by a forager, a finite abundance of resources, and technological limitations on potential rate of return. Decisions must be made about where and when to forage, which group members should go, what is transported, and what is discarded. MSA populations had the capabilities to create hafted hunting technology and complex toolkits [15, 30, 31] and how they structure foraging tasks is indicative of how these groups perceived resource availability, foraging boundaries, and landscape risks [32, 33]. Since evidence for hafting and hunting technology has implications for what technological and cognitive behaviors are attributed to MSA foragers [34], then factors that may influence the discard and archaeological visibility of these technologies on the landscape needs to be understood [35, 36]. Strategies of technological organization that emphasize serial replacement of broken and worn tools leads to variable discard locations across the landscape rather than focused retooling events at residential camps [37]. Basing our understanding of the evolution of technological systems from individual, highly visible archaeological assemblages may make certain innovations invisible by restricting the amount of behavioral variability being sampled. By incorporating a sample of technological wear traces across the landscape, hypotheses about the diversity of hunting technology, toolkit organization, and landscape use strategies can be tested. However, methodological tools for identifying variability in tool-use are needed in order to generalize about the nature of MSA technological landscapes.

This study provides a quantitative method for inferring complex histories of stone tool use and discard through a best-fit modeling approach to comparing archaeological edge damage distributions with experimental damage patterning. It presents a methodological improvement to the edge damage distribution method used previously [22, 38, 39] because it includes new experimental data and a more sophisticated model-fitting statistical analysis. With this method, the published patterning in MSA lithic points from Kathu Pan 1 (KP1), Pinnacle Point Cave 13B (PP13B), and Die Kelders Cave 1 (DK1), South Africa, are reanalyzed and multiple edge damage processes are inferred with the primary processes for each assemblage identified. This study provides a quantitative method for identifying behavioral and post-depositional edge damage formation across MSA assemblages, and in doing so, also provides a useful tool for addressing landscape scale adaptations of prehistoric hunter-gatherers.

## Background

Although stone tools are the most common surviving artifact from most sites, drawing behavioral inferences from them is not straightforward. Lithic classification and description are frequently presented as behavior, and subjective naming conventions seem to imply behavioral justification (e.g., “handaxe”, “scraper”). Much less is known about stone tool function and variability than their nomenclature implies [40].

Existing methods use micro- and macroscopic features on tool edges, tool morphology, and residue traces to make statements about past tool function [41–48]. Use-wear analysis identifies traces of microfractures, polishes, and residues that are argued to have been generated by use-action of certain configurations of tools being applied to varying materials. Lithic use-wear analysts create experimental collections of tools that consist of a variety of raw-materials, hafting arrangements, and use-intensity that are deemed analogous to the time period and archaeological technology under investigation [49]. Use-wear analysts then use a combination of polishes, microscopic linear impact traces (“MLITs”), “bright spots”, and edge scarring/dulling to infer the life history of a tool by comparison with observations from the experimental collection. This analogical approach emphasizes the size of the experimental assemblage and the experience and training of the analyst to generate archaeological data of tool function [50]. Within this method, the ‘confidence level’ is assigned by the analyst; whether they feel they have “poor” confidence or “high” confidence in their own interpretation (e.g., [51]). Use-wear analyses are often criticized for being too subjective and blind-test results have cast doubt on aspects of functional interpretations due to substantial inter-observer variation [52–54]. However, some researchers have achieved high scores on blind-tests [45, 55] and new methods are being developed to make quantitative interpretations of microscopic wear traces, [44, 56, 57].

Although the impact of post-depositional processes is not often explicitly addressed, behavioral interpretations of stone tool function are complicated by the effects of taphonomic processes on artifact surfaces and edges. Typical use-wear analyses exclude flakes that appear weathered or rolled, as are flakes from “disturbed” contexts, but the assemblage patterning is rarely described and the criteria for establishing contextual integrity are rarely made explicit [40]. Taphonomic damage is often claimed to be ‘random’ [58, 59], but statistical methods for differentiating patterned distributions are lacking. Historically, taphonomy is concerned with the study of how an organism transitions from the “biosphere to the lithosphere” [60], but has taken on a more general definition of how natural processes influence the burial of artifacts at multiple scales of observation [61]. Stone tools are the most common surviving artifact from most Pleistocene archaeological contexts, and are subject to the same processes of burial as faunal remains. Trampling, turbation, and transport are common post-depositional processes influencing the preservation of stone tools and their edge modification [62]. Therefore, a more systematic framework is needed for identifying assemblage-scale input of both behavioral and taphonomic edge damage processes.

Abundance of post-depositional tool damage formation is directly related to the degree of artifact disturbance [63, 64]. Patterning on less intensively trampled stone may be more ambiguous than heavily trampled tools (see Table 5 in [65]). Trampling edge damage can produce small regions of randomly placed edge wear, or substantially alter edges depending on exposure to disturbance processes. Morphologically, taphonomic edge damage is often described as elongated scars [59] that are dispersed along flake edges [66, 67], but occasionally cluster similar to retouched tools [68, 69] or hafted tools [70]. Shea and Klenck [63] and Pryor [58] found that trampling scars could be broad and clustered or narrow and isolated depending on the intensity of trampling and frequency of scars. Pryor [58] shows that lithic artifacts trampled on sandy surfaces can produce short, broad, randomly placed scarring, whereas loamy surfaces can produce more elongated and clustered edge damage scars that can mimic behavioral processes. Multiple studies have concluded that no individual scar can be a diagnostic clue towards inferring tool function, and that their constellation of characteristics along tool edges provides more meaningful interpretive information [22, 71].

One method recently advocated by Bird et al. [22], and adapted by Schoville [21, 39], Schoville and Brown [72], and Wilkins et al. [15, 73] utilizes the assemblage distribution of edge damage on archaeological tools quantitatively compared to experimental edge damage distributions. In these studies, instances of edge damage scars along the edge are mapped onto the artifact images in GIS, and then aggregated by assemblage to create summary distributions. Bird et al. [22] analyzed the distribution using polar statistics around the average midline of the artifacts, whereas Schoville [21] analyzed the distribution relative to the base and tip of each point. In both studies, the archaeological distributions were compared to a random, or uniform distribution of edge damage to argue that the edge damage was unlikely to be of taphonomic origin. Schoville and Brown [72] advanced this methodology further by demonstrating how experimental populations of edge damage could be compared to archaeological samples through hypothesis testing in order to make more specific behavioral inferences. Within this approach, the confidence level is relative to the statistical significance achieved and the probability of rejecting the null hypothesis when it is true.

The assemblage edge damage method has recently been challenged by Rots and Plisson [50]. In their view, function can only be established by observing multiple wear traces on individual archaeological tools that can be linked to a “large” referential collection (but see [74]). To establish projectile function, Rots and Plisson argue that multiple “diagnostic” traces must be observed on an individual tool that are suggested to be indicative of projectile function. Additionally, Rots and Plisson [50] argue that post-depositional damage cannot be understood within an assemblage of tools because there is no way to sort the “blur” of taphonomic edge damage from behavioral patterns. Wilkins et al. [73] argue that at an assemblage scale, post-depositional damage is distributed differently than behavioral damage, which allows it to be statistically differentiated. Assemblage scale analyses allow for quantification and statistical evaluation of archaeological patterning to contextualize behavioral meaning in ways that individual artifact approaches cannot [75].

In this study, edge damage distributions from experimental processes are compared to the published edge damage distributions from KP1, PP13B, and DK1 using a model-fitting approach. This technique has the advantage of linking observed archaeological patterning to varying combinations of experimental traces in order to infer the goodness of fit between experimental and archaeological distributions. The maximum likelihood approach provides the best possible model out of all combinations given the currently available data [76], thereby identifying the contributing processes that most likely influenced archaeological edge damage formation.

## Methods

Generating statistically meaningful experimental populations of lithic edge damage that can be used to infer prehistoric behavior from archaeological distributions is central to this study. Since any behavioral input to edge wear occurred in minutes or hours and post-depositional processes have been acting on artifacts for thousands of years, the first step in analysis must be testing whether the patterning, or lack thereof, is consistent with taphonomic processes rather than behavioral tool use. Two common post-depositional processes that influence artifact movement are trampling and fluvial saltation. Therefore, experimental samples exposed to each of these processes were generated.

The next step is to generate samples the represent behavioral processes. Two behaviorally meaningful uses of stone tools are as butchery cutting tools and as armature tips. Although stone tools can be, and likely were, used for a wide range of tasks [77], these two functional categories are frequently juxtaposed in MSA studies. Some studies emphasize tools used for cutting tasks [20, 23], others emphasize their use as armatures [11, 78, 79], and ethnographically points were used as both [19]. Stone tools have been used for general cutting and butchery purposes since the origin of the archaeological record [80, 81]. However the landscape variability in this behavior is not well known even in later periods such as the MSA. These two tasks reflect differences in where extractive behaviors occur on the landscape because armatures are more frequently discarded on the landscape (i.e., near kill sites [82]) whereas generalized cutting tools may be discarded more frequently in residential sites either individually as they wear out or during retooling prior to logistical forays [36, 83] because tools are discarded when exhausted in a serial fashion (e.g., page 38 in [36]). Future work will expand the range of variation in tool wear distributions, but the scope of this study is on the edge damage patterning created by trampling, tumbling, butchery, and spear-tipped armatures.

### Site Overviews

Three archaeological assemblages have previously been analyzed using the edge damage distribution method. These sites sample across the landscape from both coastal and interior locations, across site context from both cave and open-air contexts, and throughout the temporal range of the MSA. By comparing these published assemblages to new experimental processes with more sophisticated modeling technique, the existing archaeological inferences can be more critically evaluated. All material was analyzed with permission from the relevant curating institutions, and no permits were required for any aspect of the described study. Specimen numbers provided in S1 File.

#### KP1

**Fig 1:**
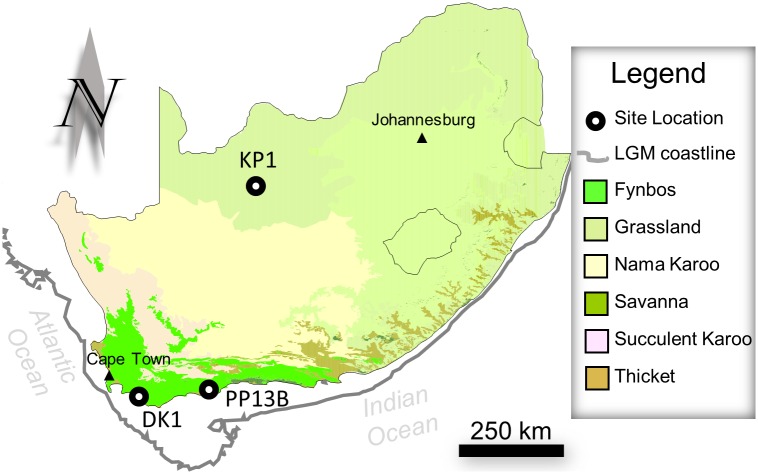
Map of site locations and vegetation regimes in South Africa.

Kathu Pan 1 is a filled in sinkhole, or doline, located in the interior Northern Cape of South Africa (27° 39’ 50”S, 23° 0’ 3”E) ~ 5km northwest from the town of Kathu [84–86] in a savanna grassland environment (Fig 1). The site was originally excavated beginning in 1978 by Beaumont [87] who identified an ESA-Fauresmith (or early MSA)—MSA sequence that includes stone tools, ochre, and a limited amount of faunal remains [85, 86]. The sample of points from Stratum 4a used here was reported by Wilkins et al. [15] and Wilkins and Schoville [38], and has an average age of ~500 ka from combined ESR and U-series dating. Lithic material from KP1 is curated at the McGregor Museum, Kimberley, South Africa and was analyzed by JW with all relevant permissions. Artifacts in stratum 4a are concentrated in two vertically oriented spring vents that is truncated by cross-cutting stratum 3 that has an OSL age of 291 ka ± 45 [85]. Most points are manufactured from banded ironstone formation, but some chert and quartzite points are included as well.

#### PP13B

Pinnacle Point is located on the south coast of South Africa (Fig 1), approximately 10 km from Mossel Bay (34° 12’ 28”S, 22° 5’ 23”E). Kaplan [88] surveyed the coastline along Pinnacle Point prior to the development of a golf course and identified 15 coastal caves and rockshelters with archaeological deposits. These caves are eroded into the quartzitic headland of the exposed Skurweberg formation of the Table Mountain Sandstone Group [89]. Recent multi-proxy dating methods have shown that the caves formed at least 1.1 ma [90]. The caves were sequentially numbered from east to west, and excavations at three of these caves have recovered an extremely well-dated sequence of MSA occupation from 164–90 ka at PP13B, 90–50 ka at PP5-6, and two ephemeral occupations between 130 and 120 ka at PP9 [91]. Excavation methods are described by Marean et al. [89]. Cave 13B contains fauna, shellfish, typical MSA stone tools, and ochre artifacts that have been OSL dated to ~162–90 ka [92]. Three areas of the cave were excavated—the Eastern Area, Western Area, and “Lightly Cemented” MSA (LC-MSA) deposits [93]. Points from PP13B were previously analyzed and reported by Schoville [21], which forms the sample that is used here. Lithic material from Pinnacle Point is curated at the Diaz Museum, Mossel Bay, South Africa and was analyzed by BJS with all relevant permissions. This study differs by dividing points into two stratigraphic groupings by Marine Isotope Stage 6 (195–130 ka) or 5 (130–80 ka). Today, Pinnacle Point is coastal, but at times during glacial periods the coastline was nearly 100 km away [94]. However, the abundance of shellfish during many MIS 6 occupations would suggest the coastline was relatively close even during these glacial phases of occupation [94, 95]. The majority of points are manufactured from quartzite available in the local Table Mountain Sandstone outcroppings, or as cobbles in nearby beaches or raised cobble beds [28, 96].

#### DK1

Die Kelders 1 is situated ~10 m above the Atlantic Ocean near the town of Gansbaai (34° 32’ 46”S, 19° 22’ 36”E) on the south coast of South Africa (Fig 1). Excavations at DK1 were initiated by Schweitzer in the early 1970s targeting the extensive LSA deposits [97]. In the early 1990s, research resumed at the site in order to expand the MSA artifact collection, explore the paleoenvironmental context of the cave sequence, and understand the geologic contexts [98, 99]. Extensive fauna, stone tools, and shell have been excavated from the MSA layers [97, 98]. The majority of the lithic raw material is quartzite, however quartz, silcrete, and chert are also present. From top to bottom, the MSA at DK1 is in Layers 6–16, with even numbers generally having greater anthropogenic input than the odd layers [100]. There is a major shift in raw material towards silcrete beginning in Layer 10, and culminating in Layer 12 [101, 102]. A similar shift occurs at other MSA sites along the south coast, however this shift is usually associated with the appearance of Still Bay or Howiesons Poort technologies which are absent at DK1 [101, 102]. ESR and OSL ages for DK1 situate it at ~70 ka ± 10, roughly concurrent with these technologies and raw-material shifts elsewhere [103]. For this study, the DK1 layers were grouped into the early layers associated with this raw material shift (Layers 10–14), and the later layers that are composed of quartzite (Layers 6–9). MSA layers 6–9 have abundant marine mammals and shell (in micromorphology), whereas Layers 10–16 have no evidence for marine shells and very few marine vertebrates, and may have been deposited during a period of lowered sea-level [104, 105]. Lithic material from DK1 is curated at the Iziko Museum, Cape Town, South Africa and was analyzed by BJS with all relevant permissions.

### Data Acquisition

**Fig 2:**
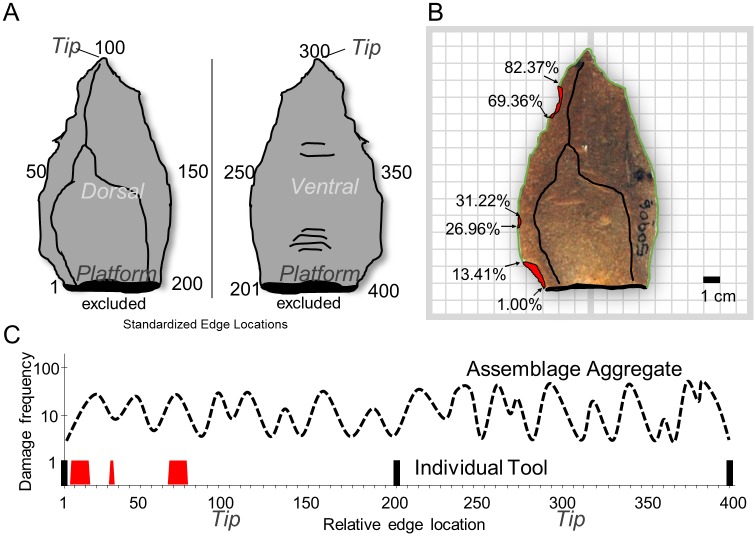
Edge damage data collection. A) Tool perimeter is divided by left and right sides based on maximum distal extent. Each edge is then divided into 1% intervals based on edge perimeter between the platform and distal maximum. In this way, each side contains 100 possible locations where damage could occur, regardless of size differences. Dorsal contains damage locations 1–200 and ventral contains damage locations 201–400. B) Photographs are taken from dorsal and ventral views onto a grid, then georeferenced and the outline digitized and edge damage scars traced. Presence or absence of edge damage in 1% intervals is calculated from the polygon shapefiles. C) Edge damage occurrences are aggregated (dashed line) based on the distribution of edge damage around individual tool edges (red histogram bars). The red bars indicate damage on point shown in B that would then be aggregated with other points from the same assemblage or experiment group, and thick black bar indicate platform-adjacent locations.

Every tool was photographed on the dorsal and ventral surface with a DSLR camera with macro lens onto a grid using a portable light tent to ensure uniform clarity and color correction ability. The camera was mounted to a tripod with adjustable horizontal arm to ensure stable imagery, and every photograph is taken from an appropriate height above the artifact to minimize image distortion. Digital images were then georeferenced in ESRI ArcGIS 10.2 using a background grid for landmarks (Fig 2B). For every specimen a shapefile was created for both the dorsal and ventral side and it contains the specimen number, damage classification codes, and damage metrics. A polygon is then traced around each specimen. Shapefiles available in online data repository (https://figshare.com/s/f1cfd33a076f080a2bbc).

Every specimen was then analyzed for macroscopic fractures under a binocular stereomicroscope with strong incident lighting. A maximum of 30x magnification was used to identify the nature of damage. Using the digitized image as a guide, individual edge damage occurrences are traced around individual damage scars by visually identifying on the imagery the outline of edge damage identified under microscopy. Each damage polygon is categorized based on visual morphology (e.g., crushing, snap, rounded—following Tringham et al. [59]); and retouch is defined as continuous invasive edge modification with negative bulbs of percussion.

Each shapefile was standardized based on the location of damage from the platform to tip (Fig 2 and S2 File). An Excel template was then used to calculate total edge length and scale to 100. This removes the effect of size differences so that edge damage locations along the tool edge are all relative to the standardized tool edge length between the platform and tip on that edge (Fig 2A). The resulting data matrix consists of each tool face and edge (i.e., dorsal left edge of specimen 305308) and 100 columns where the presence/absence of edge damage is expressed as either “1” (present) or “0” (absent). For instance, if there was an edge damage scar that was 3% of the total edge length centered halfway up the edge, then columns 49, 50 (the exact midpoint), and 51 would have a value of “1” for that edge, while the remaining 97 locations would have a value of “0”. These damage counts can then be totaled for the location (sum of all damage that occurs at a single relative location), for a tool edge, for a complete tool, for a stratigraphic level, and higher scales of analysis. For example, if there were 100 tools, and every edge of every tool was completely damaged, then the total amount of damage possible would be 100 tools * 4 edges * 100 locations each edge could possibly be damaged in, = 40000. In reality the amounts of damage are lower than this, but this illustrates how damage counts may be totaled, and undamaged areas excluded (S2 File).

One difference in the study presented here compared to previous studies of assemblage edge damage analysis, is that all edges are included and analyzed simultaneously. Each tool consists of four edges ordered starting at the dorsal left edge next to the platform, around the distal to the dorsal right edge next to the platform, and continuing around the ventral perimeter in the same fashion (Fig 2 and S3 File). This allows a single row of data to be associated with each point, and then summarized by assemblage or experiment, while still retaining the overall distribution of edge damage around the complete tool. For KP1, Wilkins and Schoville [38] analyzed only the ventral distribution of edge damage because the dorsal was not statistically different from random. Here, we include the dorsal distribution from KP1 so that more nuanced inferences of post-depositional processes can be inferred using the complete distributions.

### Model-Fitting Approach

Human behavior is extremely variable, and there are more possible combinations of tool types, hafting arrangements, and tool uses than in any experimental collection. Given variability in assemblage composition it is expected that many sites may be significantly different from all experimental populations. Therefore, the experimental distributions of lithic edge damage are treated as models and assessed against the archaeological patterning, and the best model can be quantitatively arbitrated using a model selection inference criterion called the Akaike information criterion (AIC), which not only accounts for the increase in fit with added parameters (e.g., multiple edge damage distribution process combinations), but also penalizes a model for having added parameters without sufficient increase in the explained variance thereby preventing over-fitting [106]. Results of this maximum likelihood approach provide the best possible model given the currently available data, and makes them comparable among assemblages [76].

This statistical procedure is an advance over previous work that relied solely on hypothesis testing because it is multivariate, less sensitive to low sample sizes, and less susceptible to Type II errors [76, 107]. The stepwise regression models used here were analyzed in JMP Pro 12 statistical analysis software using a forward stepping (additive) procedure where the term with the lowest *p*-value is added first, and then subsequent terms are added and removed until the best model is found. The best model is one with the lowest value for AIC, but if the change in AIC (ΔAIC) is <2, then the models are considered equivalent and the model with fewer parameters is selected [106]. Each term is given equal weight to enter the model, but will explain different amounts of the residual error. In other words, a best model with multiple terms (e.g., armatures + trampling) will be selected based on the overall improvement in model fit, but the terms will explain different amounts of the variance in observed archaeological edge damage patterning.

Each edge damage distribution was smoothed using a loess non-parametric generalization with alpha smoothing set at 0.15 in order to minimize the influence of extreme values in the dataset due to random error [108]. The archaeological and experimental distributions are available in S3 File. The analysis proceeds in order of increasing specificity so that more general patterning is diagnosed first, and more specific models incorporated second. In the first phase, the result from fitting a single parameter to the archaeological data is presented using the smoothed armature-tips, cutting tools, trampling, and tumbling distributions. These results provide an indication of what experimental process is most consistent, or explains the greatest amount of variability in archaeological edge damage patterning. Once this result is presented, a more specific model fitting algorithm sequentially adds and subtracts parameters until a model with the lowest AIC is reached for all possible model combinations. With n-parameters, the best fit model can contain anywhere from 1 to n variables. When n>1 in the full-set model, the R^2^ value will always be lower than the single-fit model. On some models, the R^2^ values are low, even though the likelihood procedure identified it as the best-fitting model. Highly variable data can produce low R^2^ values, even though a significant trend has been fit. Given the multitude of processes that can influence edge damage formation, it is unlikely to find a perfect fit. However, the model-fitting procedure identifies the most likely process or combination of processes given the currently available data. Therefore, the model that is chosen is selected based on quantitative criteria, but is subject to further refinement in the future as additional experimental processes are added as potential terms for the model fitting. The methodology outlined here should serve has a baseline for future likelihood approaches to lithic use-wear and functional analyses.

### Experimental Armature Sample

**Fig 3:**
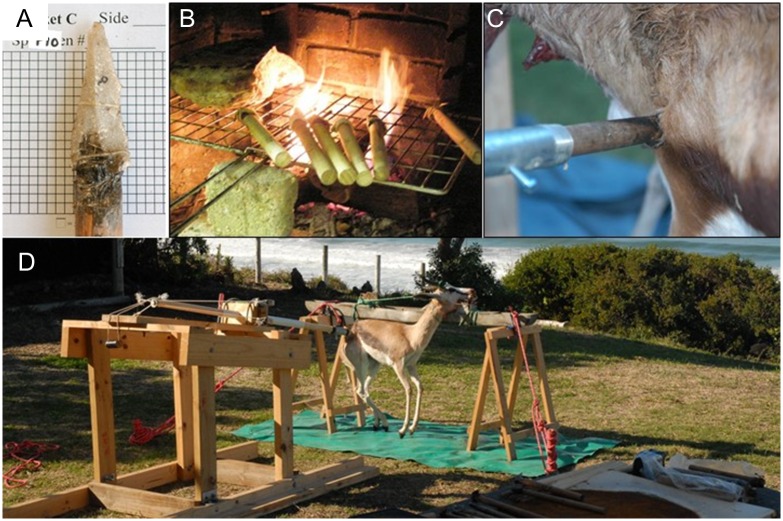
Spear armature setup. A) Quartzite point hafted to wooden dowel. B) Points drying near heat source. C) Point lodged in carcass after being fired. D) Calibrated crossbow setup.

A calibrated crossbow was constructed following Shea et al. [109] to create experimental patterns of edge damage from thrusting spear use (Fig 3). Experimental points similar to those recovered from MSA assemblages were replicated using quartzite local to the Pinnacle Point caves (n = 61) as well as silcrete (n = 3). Each convergent flake was hafted to a wooden dowel using a combination of *Acacia karroo* mastic and sinew (Fig 3A). The experimental points were initially thrust once and then examined for edge wear. Each surviving point (i.e., still forming a point) was thrust until a catastrophic break occurred, up to a maximum of six trials. Points were thrust a total of 150 times for all points (mean = 2.34 thrusts per point). The crossbow was calibrated to 28 kg of draw force and kept constant for each replication. This preliminary sample was initially reported in Schoville and Brown [72], while the sample here includes an additional two unpublished springbok experiments and 42 additional unpublished points. Four springbok carcasses (*Antidorcas marcupialis*) culled from a private game farm near Oudtshoorn, South Africa, were purchased commercially through Lizelle Bezuidenhout. Livestock were obtained directly from a supplier to local butcheries for the purpose of private use and consumption as part of routine food supply channel, and was not subject to IACUC review at ASU.

Experimental ironstone armatures were initially reported by Wilkins et al. (2012) and followed essentially the same protocol using the same calibrated crossbow. The main difference in experimental protocol is that the ironstone points were shot at the target until they broke, with no limit to the number of shots required. These experimental armatures will only be used to fit the KP1 material, as ironstone is not readily available as a raw material near PP13B or DK1.

### Experimental Butchery Sample

**Fig 4:**
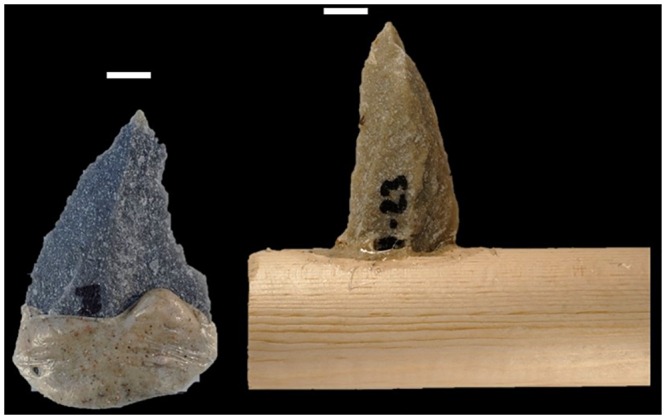
Butchery tools. Left, quartzite with mastic; right, quartzite in slot haft with mastic. White bar is 1cm.

Three domestic pigs (*Sus scrofa domesticus*) were purchased as livestock for the purpose of private use and consumption in Maricopa County, Arizona. Prior to this study, swine were obtained and slaughtered according to all pertinent regulations for humane livestock slaughter of swine, including initial dispatching with a large caliber firearm (Maricopa County A.R.S. § 3–2016). Since livestock were obtained through routine food supply channels, this study was not subject to IACUC review as would be the case from obtaining road kill or veterinary cadavers. The pigs were butchered with quartzite points, flakes, and blades (only points reported here). These experiments were all performed by an experienced butcher, hunter, and licensed journeyman farrier with extensive knowledge of ungulate anatomy. This single butcher was used to keep butchery technique constant and remove inter-experimenter variability in stone tool use. The butcher was instructed to use tools in however manner felt comfortable and was allowed to wear a glove for hand protection. As soon as the butcher felt a tool was “too dull”, it was immediately retired and a new tool was selected by the butcher. A total of 60 silcrete and quartzite tools were prepared for the butcher, of which 20 were points. In addition to unhafted tools, two basic hafting styles (Fig 4) were made using mastic obtained from commercial grade acacia gum (“gum Arabic”) following traditional Australian *Leilira* blades [110, 111]. While there are numerous possibilities for hafting methods, these two strategies involve the fewest techno-units [112], are well known from the ethno-historic record, and serve as a starting point for the assemblage edge damage method. The powdered resin was mixed with water over low heat on a stove using an initial ratio of 2.5g resin, 2.5g water, and 1g sand, following the recipe provided by Zipkin et al. [113]. The mixture was allowed to air dry until tacky, then applied to stone tools. Ten quartzite MSA points were lodged into 20cm long hard-wood handles using a slot-haft, then reinforced with the mastic mixture. A small mass of mastic was applied to point bases to form a handle following images in Tindale [110].

**Fig 5:**
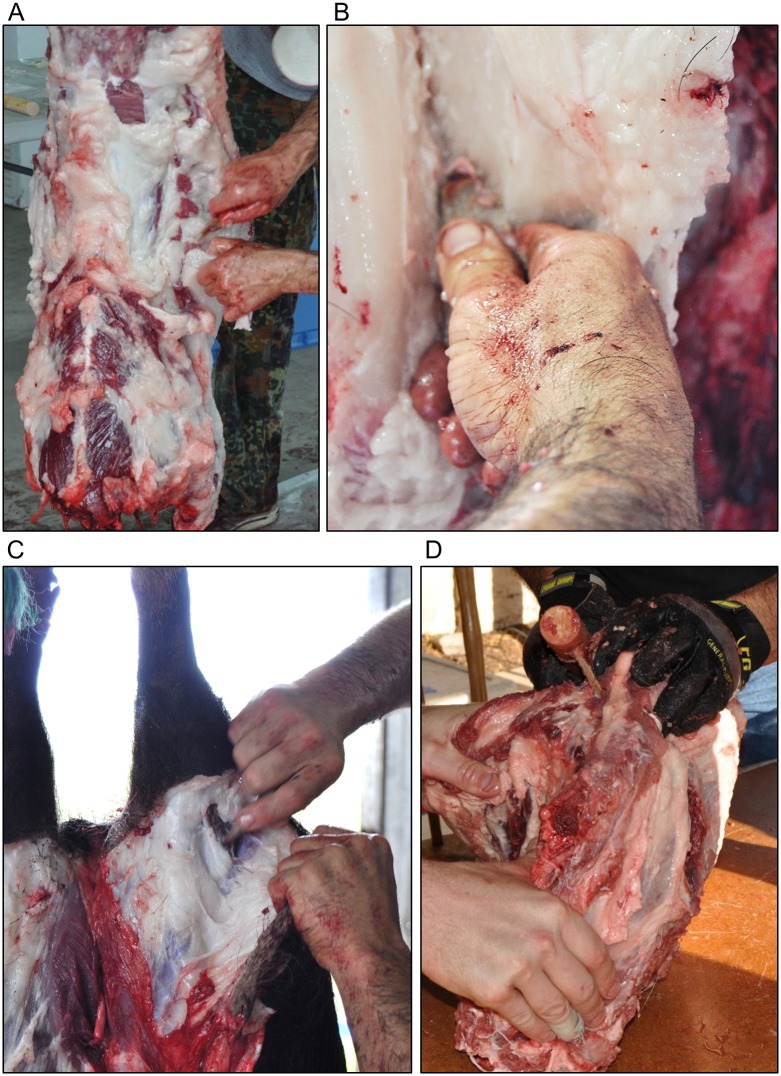
Butchery experiment completed with quartzite points. A-B) Handheld tools during field dressing. C) Handheld tool during initial skinning. D) Hafted tool during defleshing.

The butchery was divided into two stages that represent different activities that were likely to occur in different places on the landscape. The first stage was the initial “field dressing”, where the animals were eviscerated (Fig 5A and 5B), skinned (Fig 5C), and disarticulated into manageable units. The second stage of “defleshing” involved cutting the meat from around the bones and reducing conjoined elements into parts that could be efficiently managed while cooking shown in Fig 5D. Combined, the two stages form the general butchery distribution while more specific models incorporate the two stages, field dressing and defleshing, separated.

### Experimental Trampling Sample

After being discarded and prior to burial, stone tools are vulnerable to being stepped on by humans and animals. There have been numerous studies directed at understanding the effects of trampling on stone tools [58, 63, 64, 66–69, 114]. Several factors have been shown to influence the production of trampling damage to flakes, including raw material, the duration of trampling, the density of artifacts, and how compact the sediment is. These factors also influence the spatial disturbance of artifacts.

**Fig 6:**
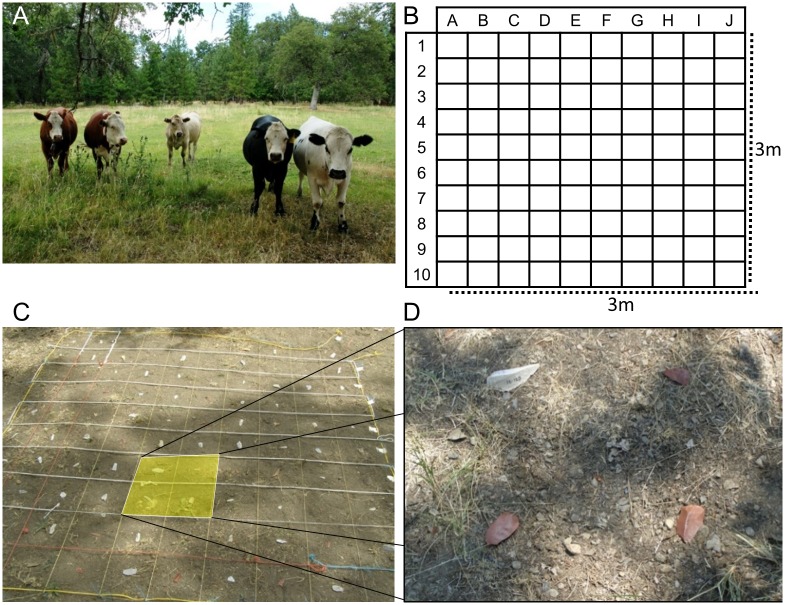
Trampling experiment layout. A) Cattle preparing for trampling. B) 3 x 3 m grid layout used at each site, with tools laid out alternating dorsal and ventral side-up. C) String used to lay out grid on the ground with four cells highlighted. D) Close-up view of highlighted cells showing tools prior to the trample experiment.

Unlike studies of trampling that tend to be short, focused, intentional trampling events [63, 114–116], often with human tramplers, for this experiment a long-term study site was used. Artifact burial is likely a process on the order of weeks or months (if not years), therefore a long-term study site is more applicable to the archaeological record than 30 minutes of human trampling. These experiments were performed with the permission of Keith Groves at Alpen Cellars, a vineyard in Northern California (41° 0’ 43”N, 122° 36’ 42”E) that also maintains a small domestic stock of four cattle, two unshod horses, and is home to a variety of wild deer, bear, and small mammals (Fig 6A). Three different contexts were selected for trampling sites based on the anticipated degree of animal activity in uncultivated regions of the vineyard. The high-intensity site is a coral used periodically to restrict the movements of the cattle prior to being transported off-site. Horses and cattle are periodically fed in the coral, attracting their presence frequently. The ground surface in the coral is barren, and the sediment is soft clayey-silt, that turns into mud during storms. Although substrate has been shown to influence the abundance of damage that occurs due to trampling, prior studies have not found significant differences in the distribution of damage along tool edges due to substrate [58]. In other words, there is little reason to suspect that substrate should influence where along the tool edge damage is more likely to occur due to trampling. The medium-intensity site is adjacent to a cattle trail that leads to the coral, located on a small grassy field between two water culverts. Animals would pass through this area, and occasionally graze on the grasses, but it is not a large area nor a constrained area in which intensive activities would take place. The area is surrounded by deciduous trees, and the leaf-litter was raked clear prior to laying out the flakes. The soil is a silty loam, and highly organic with grasses, roots, and weeds present. The low-intensity site is located on the edge of a large field. While the area is occasionally grazed by cattle and horses, it is a large area and no repeated concentrations of animals was anticipated. This area is a fluvial silty floodplain, mostly covered with perennial rye grasses. Some small granite and shale cobbles were noted in the area. This area was not raked clear prior to setting out lithic tools because the leaf-litter was much lighter than in the medium-intensity area.

No direct animal interaction took place, all evidence of animal traffic in the area was obtained from remote motion triggered cameras, and therefore were not subject to IACUC review at ASU. Motion-sensitive digital cameras (Primos Truth Cam 35^®^) were used to monitor activity in the three areas without interfering with normal activity on the vineyard property. A camera was placed ~2m high (above cattle height) on nearby trees, with an empty 16 GB Secure Digital (SD) Flash memory card. These cameras are rated for 6 month-battery life, but the batteries were changed after 3 months (October) by KSB to ensure functionality.

At each trampling site, 100 detached pieces were used consisting of 40 quartzite, 40 silcrete, and 20 quartz and ironstone flakes. A variety of shapes and sizes were used, of which 61 were points that compose the trampling edge damage distribution model. Detached pieces were laid out in a 3 x 3m grid, divided into ten evenly spaced columns (A-J) and rows (1–10) using string, so that 100 cells of equal 30 x 30cm size were created (Fig 6B). This allows each artifact to have a buffer around it to minimize contact with other tools (Fig 6C and 6D). This may be less realistic for comparisons with dense archaeological accumulations, but provides a baseline of damage patterning when tools are scattered and exposed to surface trampling. Metal stakes were driven into the corners of each trampling area to ensure recovery after 6 months of trampling. A stratified-random assignment of flakes to trampling area, column, and row was used. Detached pieces were then laid out by alternating dorsal and ventral side-up in the center of each cell (established by using a straight-edge to connect the corners and placing the flake in the center “X”). In this way, each trampling site was randomized, containing equivalent frequencies of tool shape, raw-materials, and side-up.

After 5 months of exposure (August–December), the tools were collected. A Topcon Total Station was used during recovery to piece-plot the location of each tool. Since the starting position of each tool is known relative to the corners of the 3x3 grid, starting coordinates were able to be calculated retroactively by obtaining the coordinates of the grid corners, and then offsetting for each cell. For instance, cell A1 would be in the Northwest corner of each grid, and the center of the cell is 15cm south and 15cm east of the corner coordinates. Each tool that was recovered *in situ* was piece-plotted with the total station and the side-up was recorded.

### Experimental Rock Tumbling Sample

**Fig 7:**
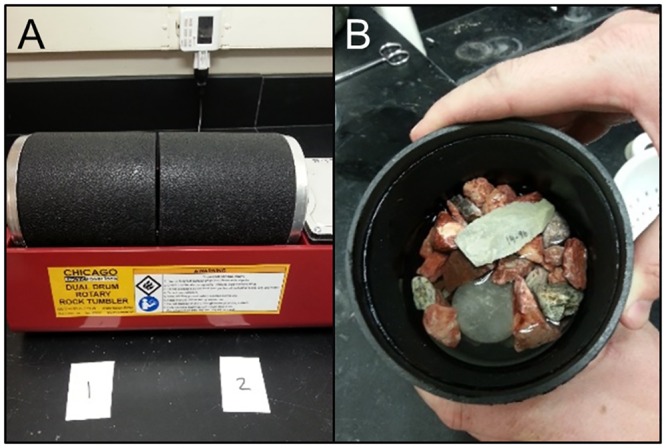
Rock-tumbler experimental setup. A) Two drums and digital timer to control tumble duration. B) Drum with water, gravel, quartz stone matrix.

Chambers [117] has shown that during flume experiments, lithic damage mainly formed during artifact saltation. A water-filled rock tumbler is often used by geologists to mimic the effects of long-term fluvial saltation in a short amount of time (e.g., [118, 119]). In this experiment a mixture of coarse gravels (avg. 26mm length), water, a quartz hammerstone, and individual silcrete and quartzite detached pieces were placed into a dual drum rotary rock-tumbler (Chicago Electric^®^ Power Tools) to simulate the impact of fluvial activity on stone tool edges (Fig 7). The mass of each barrel including water, gravel, and hammerstone was similar (Barrel 1 = 422g; Barrel 2 = 434g). Sixty tools evenly split between quartzite and silcrete were prepared for this experiment, of which 22 were points (only type reported here). After trial and error, a duration of 5 minutes was decided on, because this amount of time created some damage without completely rounding all the edges.

## Results

A summary of each experimental and archaeological sample is shown in Table 1. In terms of edge damage distribution, each experimental edge damage distribution was significantly different from every other experimental distribution (e.g., Armatures vs Trampling, Armatures vs Tumbling) using Kolmogorov-Smirnov test for distribution equality (*p* = 0.05). In the following section, results from each experimental process will be presented, followed by the results of the model-fitting for archaeological assemblages of points. Experimental tool shapefiles are available in an online repository (https://figshare.com/s/f1cfd33a076f080a2bbc).

**Table 1:** Sample of experimental and archaeological points examined for edge damage.

Assemblage/ Experiment	Total examined points	Points with edge damage
PP13B—MIS5	203	71 (35%)
PP13B—MIS6	89	16 (18%)
DK1—Layers 6–9	37	12 (32%)
DK1—Layers 10–14	50	24 (48%)
KP1^1^	106	90 (85%)
**Archaeological Total:**	**485**	**213 (44%)**
Armatures—Quartzite	64	49 (77%)
Armatures—Ironstone^1^	32	32* (100%)
Butchery	20	18 (90%)
Trampling	61	49 (80%)
Tumbling	22	20 (91%)
**Experimental Total:**	**199**	**177 (84%)**

*Shot until damage was evident.

### Armatures

**Fig 8:**
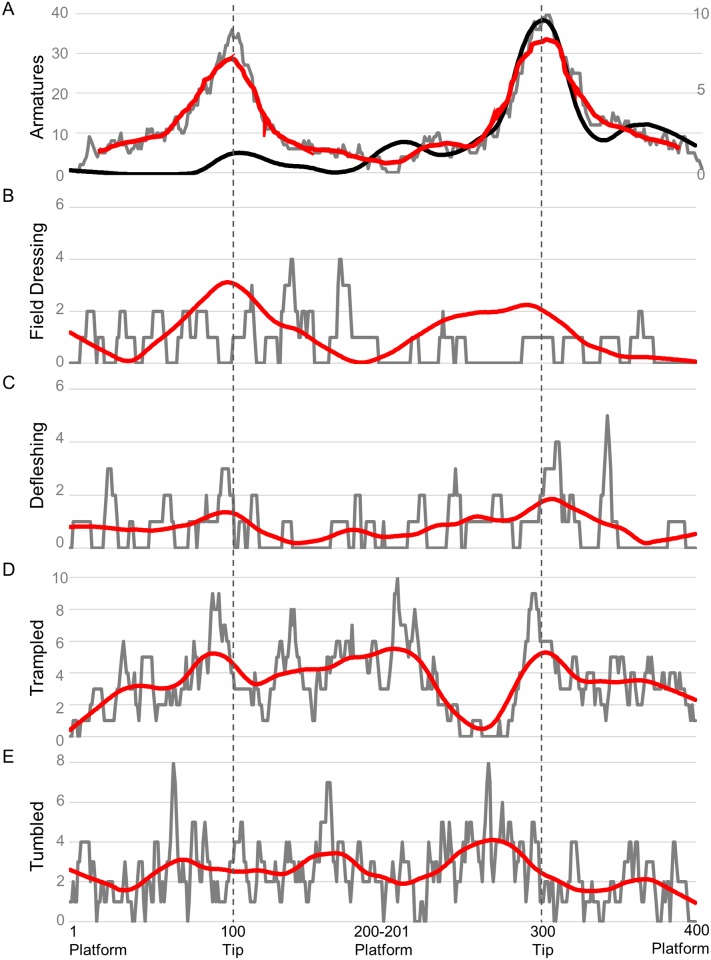
Experimental damage distributions (grey) and loess-spline (red) from: A) spear tipped armature use (black line is ironstone loess-spline scaled to right y-axis), B) field dressing butchery activity, C) defleshing butchery activity, D) long-term trampling by animals, E) rock-tumbler for five minutes.

The spear-tipped armature experiments resulted in extensive edge damage to the points, including numerous distal breaks and impact fractures, as well as hafting damage closer to the proximal end of the points The overall distribution of damage on spear points (i.e., where the damage is located on average along the tool edge) is concentrated at the tip (Fig 8A). The distribution of spear point damage along the point edge is not significantly different between the left and right sides (KS-test, *p* = 0.1613), or between dorsal and ventral faces (KS-test, *p* = 0.9963), and a slight increase near the base of points is seen, likely relating to the extent of hafting bindings along the tool edge.

### Butchery

The butcher used 20 points during butchery, of which 18 exhibited visible edge damage during analysis. The butchery experiments produced extensive damage that exhibit patterning on the utilized points. Butchery resulted in more damage on the left edge than the right, but was formed equally between dorsal and ventral faces. It is not known how handedness affects this pattern, but it is anticipated to be the opposite for a left-handed butcher [21]. Overall, there is more damage on the left edge compared to the right (χ^2^ = 12.454, df = 1, *p* = 0.0004). When split into processing activity, the left edge has significantly more damage than right on field dressing tools, (χ^2^ = 15.273, df = 1, *p* = 0.0001), while the left edges of defleshing tools have more damage, the difference is not significant (Left n = 170, Right n = 155, χ^2^ = 0.692, df = 1, *p* = 0.405). The dorsal and ventral faces do not have significantly different frequencies of damage either in aggregate (χ^2^ = 0.672, df = 1, *p* = 0.412) or when divided into processing activity (Field processing, χ^2^ = 0.312, df = 1, *p* = 0.577; Defleshing, χ^2^ = 3.769, df = 1, *p* = 0.052).

The distribution of damage created from defleshing and field processing activities along point edges are significantly different (KS-test, *p*<0.0001, Fig 8B and 8C). Overall, the left and right distributions are distributed differently (KS-test, *p*<0.0001), which holds for both field processing (KS-test, *p*<0.0001) and defleshing (KS-test, *p* = 0.0005) activities.

How handedness and idiosyncrasies of individual butcher grip and cutting motion influence the overall frequency and distribution of damage is unknown. The distributions shown in Fig 8B and 8C provide a starting point for identifying different phases of butchery processes in assemblage edge damage formation.

### Trampling

**Fig 9:**
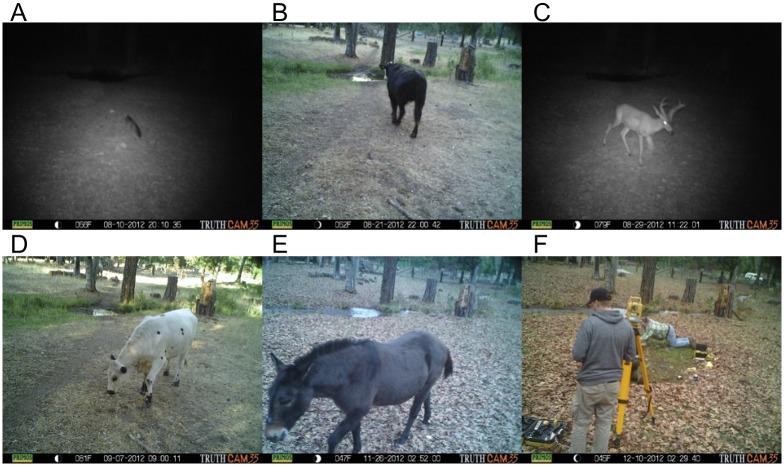
Motion camera photos from trail. A) endangered Humboldt Marten; B) cattle passing through trampling area during day; C) deer passing through trampling area at night; D) cattle lingering in trampling area; E) donkey passing through trampling area; F) Authors BJS and KSB recovering tools and piece plotting in the trampling area at the end of the experiment.

The number of images taken by the motion cameras positioned at each trampling site indicate that the corral had the greatest animal activity, but that the field had more animal activity than the trail location (Table 2). Based on the images that were captured (Fig 9), the animals tended to stay and graze in the open field for longer periods, which caused the camera trap to take more photographs. In contrast, the trail had a greater diversity of animals, but images were typically of them walking through and not lingering in that location.

**Table 2:** Frequency of motion capture images captured by trampling location.

Location	Within Field	Trail Adjacent	Within Corral
Anticipated Trampling Intensity	Low	Med	High
Total Images	2734	2147	8231
Average/day	21.7	17.0	65.3

Tools were recovered from each of the three areas consistent with expectations (Table 3)—the field had the highest recovery rate, followed by the trail, and the corral had the lowest recovery rate. At the corral, only 22 of the recovered 65 tools were able to be piece plotted because of the severity of artifact movement both vertically within the clayey mud, as well as laterally outside of the trampling grid. After trowel excavating the entire 3 x 3m grid 20cm deep, it was determined that due to time constraints a 1m perimeter around the grid would be excavated with shovels and screening through ¼” mesh. This method resulted in the recovery of an additional 43 tools, which have no post-experiment provenience. At the field and trail sites, artifacts were generally still located on the surface and very little excavation was needed. Every artifact recovered was piece plotted at these two trampling areas. Similar to the recovery rate data, the rate of artifact flipping (i.e., from dorsal to ventral side-up or vice-versa) was correlated with the expected trampling intensity. The corral had a high-degree of artifact flipping (59% of piece-plotted tools) while the field had the lowest degree of flipping (38%). Despite the difference in motion-detection photographs between the trail and field locations, it seems that the trail was subjected to more disturbance than the field. This may be because animal movement causes more damage than animal loitering, which was generally the case in the field.

**Table 3:** Recovery frequency of all artifacts by trampling location.

Location	Total Station Plotted (% of total recovered)	Total Recovered (% of start)	Flipped from Start (% of plotted)
Field	95 (100%)	95 (95%)	36 (38%)
Trail	87 (100%)	87 (87%)	38 (44%)
Corral	22 (34%)	65 (65%)	13 (59%)

The aggregate distribution of edge damage on trampled flakes, blades, and points is not significantly different from a uniform distribution (KS-test, *p* = 0.791). Separately, the distribution of damage on blades is also not significantly different from uniform (KS-test, p = 0.497), however flakes (*p* = 0.012) and points (*p*<0.0001; Fig 8D) are significantly non-uniform. This is consistent with recent findings from McPherron, et al. [116] who found a significantly non-random distribution of edge damage on trampled flakes, which they relate to uneven distribution of edge angle on flakes (and likely points) in particular.

In terms of side-up frequency and damage formation frequency, damage forms more readily on the upward facing surface. Table 4 shows this pattern for the three trampling intensity areas. At every location, when dorsal was face up, the dorsal face had the most damage, and when ventral was up, ventral had the most damage (Dorsal up, χ^2^ = 71.426, df = 1, *p* = 0.0001; Ventral up, χ^2^ = 7.392, df = 1, *p* = 0.0066). Overall, more damage formed on the dorsal surface (χ^2^ = 12.032, df = 1, *p* = 0.0005) despite more tools having been plotted ventral up post-trampling across the three trampling areas (Dorsal up = 88, Ventral up = 92) and more flakes flipping from dorsal to ventral (n = 36) than from ventral to dorsal (n = 35).

**Table 4:** Edge damage frequency by trampling location and recovery ‘face up’.

Trampling Location	Total dorsal up		Total ventral up	
	Dorsal	Ventral	Dorsal	Ventral
Corral	436	248	351	408
Field	321	207	252	276
Trail	375	309	598	654

### Rock Tumbling

After exposing points to five minutes of tumbling in a rock-tumbler, extensive damage across all tool types was observed. Although some tools had nearly continuous damage around the tools, the damage tended to be very shallow. The aggregate distribution of edge damage on tumbled flakes, blades, and points is not significantly different from a uniform distribution (KS-test, p = 0.3669). However, the distribution of damage on points alone due to tumbling in a rock-tumbler is significantly different from a uniform distribution (KS-test, *p* = 0.0334), also consistent with variability in edge angle unequally influencing the damage distribution on points (Fig 8E).

### Model Fitting

The model fitting procedure is first tested by using the experimental distributions generated above to fit known distributions of edge damage. To do this, each of the experimental distributions are included in a stepwise regression model and the process(es) that is most consistent with the known edge damage distribution is identified. In Table 5, the four experimental edge damage distribution models of known causal agency (i.e., quartzite spear-tip armatures, combined trampling areas “trampling”, butchery processes combined “butchery”, and rock tumbler “tumbling”) were used as parameters for six independent known distributions consisting of the experimental ironstone spear points published by Wilkins et al. [15], and five distributions of randomly generated edge damage of size n = 10000, 1000, 500, 100, and 50. The random distributions were created by randomly sampling from a uniform distribution of edge damage (i.e., each location along edge had equal probability of damage) n-times with replacement.

**Table 5:** Testing model fitting procedure with known distributions of edge damage.

Assemblage	Best-fit Parameter	AICc	R^2^
Experimental Ironstone Spears	Quartzite Armatures	1930.96	0.332
Random (n = 10,000)	Trampling	2942.17	0.001
Random (n = 1000)	Tumbler	2088.91	0.002
Random (n = 500)	Tumbler	1782.70	0.002
Random (n = 100)	Trampling	1117.79	0.036
Random (n = 50)	Trampling	907.00	0.001

As anticipated, the experimental ironstone spear tip distribution is best fit by the quartzite armature distribution (R^2^ = 0.332). The remaining unexplained variance is likely due to raw-material differences between the fine-grained ironstone and relatively coarse-grained quartzite. For the random distribution assemblages, we would not anticipate behavioral processes to fit the distribution, nor would the trampling and tumbling distributions necessarily. In fact, we find that for each random model (n = 10000, 1000, 500, 100, and 50), post-depositional processes are the single best-fit variables, but generally only account for less than 1% of the variability. Importantly, these examples highlight that this procedure does not fit behavioral causal agents to random trace patterning, but also that the closest behavioral processes (quartzite armatures) best fits the ironstone armature distribution.

#### General processes

**Fig 10:**
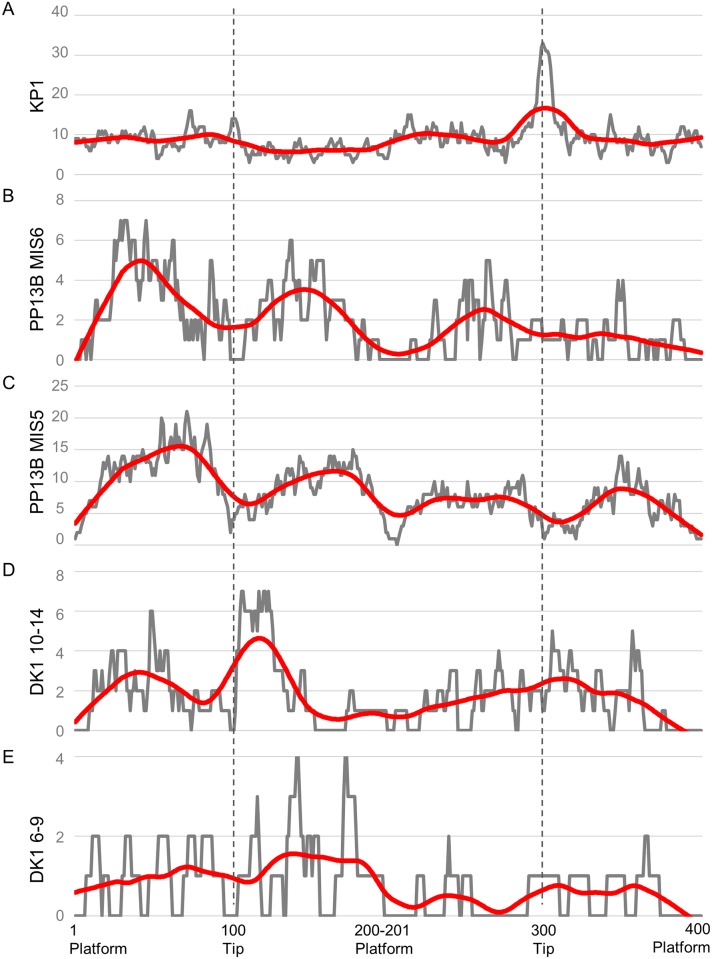
Temporally ordered (oldest to youngest) archaeological edge damage distributions (grey) and loess-spline (red) on points from A) Kathu Pan 1, B) the MIS6 layers at PP13B, C) the MIS5 layers at PP13B, D) layers 10–14 at DK1, and E) layers 6–9 at DK1.

The distribution of edge damage on points from each of the archaeological assemblages analyzed are shown in Fig 10. The single best-fit parameter of experimental processes for each archaeological assemblage is provided in Table 6. These are the most general category of edge damage formation given the currently available models, and would be most consistent with prior studies that utilized the assemblage edge damage distribution method [15, 22, 72]. Working from the most general to more specific experimental processes establishes whether the archaeological edge damage distribution patterning is best fit by armatures, cutting, or taphonomic processes. This places the more specific experimental processes in context. For instance, identifying defleshing processes as significantly explaining variation in one assemblage of archaeological edge damage has different meaning if the general pattern is most consistent with post-depositional damage compared to butchery.

**Table 6:** Single best-fit general experimental parameter for each archaeological assemblage of points.

Assemblage	Best-fit Parameter	AICc	R^2^
KP1	Ironstone Armature	1410.7	0.66
PP13B MIS6	Taphonomic	1266.7	0.05
PP13B MIS5	Taphonomic	2050.1	0.07
DK1 10–14	Butchery	1020.3	0.32
DK1 6–9	Taphonomic	385.2	0.14

The single best-fit processes which explains KP1 edge damage distribution (Fig 10A) are experimental ironstone armatures (F-ratio = 454.089, p<0.001, R^2^ = 0.533), which is also the highest R^2^ value achieved for any of the archaeological assemblages. Post-depositional processes are the single best-fitting parameters for both of the PP13B MIS aggregates, although the model-fit is relatively poor (R^2^ < 0.10). This is in contrast with prior studies that compared the PP13B edge damage to random or uniform distributions. This is consistent with McPherron et al.’s [116] observation that taphonomic processes could lead to ‘patterned’ results on tools when edge angle is patterned. At DK1, the lower levels have edge damage patterning most consistent with butchery processes with a relatively high R^2^ value (0.32), while in the upper levels (6–9) post-depositional damage is the single best-fitting model parameter.

#### Specific processes

Table 7 provides the complete best-fitting model for each archaeological assemblage of points analyzed here. This permits a more nuanced inference of edge damage patterning to be made based on the presumption that multiple processes likely acted on stone points to produce the observed damage patterning.

**Table 7:** Results of model-fitting experimental edge damage distributions to archaeological points. Parameter percentage of residual sum-of-squares contribution in parentheses.

Assemblage	Best-fit Model	AICc	R^2^
KP1	Ironstone Armature (64%)+Deflesh(32%)+Tumble(4%)	1274.3	0.76
PP13B MIS6	Trample(34%)+Deflesh(30%)+Tumble(20%)+Armature(16%)	1241.1	0.12
PP13B MIS5	Tumble (46%)+Deflesh(31%)+Armature(15%)+Field(8%)	2005.8	0.18
DK1 10–14	Field(43%)+Tumble(29%)+Armature(14%)+Trample(11%)+Deflesh(3%)	961.1	0.43
DK1 6–9	Trample(47%)+Tumble(33%)+Deflesh(17%)+Armature(3%)	317.6	0.29

At KP1, The best complete model also suggests a contribution from defleshing and post-depositional tumbling. Since the distribution of experimental spear tipped armatures alone explains 66% of the observed variance in archaeological edge damage on KP1 points, the addition of defleshing and tumbled edge damage distributions explains an additional 10% of the variance.

At PP13B, during MIS6 (Fig 10B), the full model indicates a contribution from trampling and defleshing processes, with more minor contributions from tumbling and armatures. The improvement in model fit from R^2^ = 0.05 to 0.12 indicates only an additional 7% of variance in observed MIS6 edge damage is explained with three additional variables, and there is still 88% of the variance unexplained by the full model. Additional work is needed to identify an experimental edge damage process that can better account for the distribution of damage in MIS6. The assemblage of points from MIS5 (Fig 10C) includes tumbling and defleshing tools as primary processes with minor contributions from the armature and field dressing distributions. The percentage of explained variance increases from 7% to 18% with the additional parameters. This pattern is consistent with Schoville [21] who did not identify any major differences between MIS 6, late MIS 6, early MIS 5, and late MIS 5. Points exposed to primarily post-depositional and defleshing tool use is consistent with the observed archaeological edge damage from PP13B, however a significant amount of variation is left unexplained by the current model.

Points from DK1 Layers 10–14 (Fig 10D, increased silcrete layers) are best-fit by a model with every potential parameter—i.e., every parameter significantly added to the amount of explained variance and lowered the AICc by more than 2. Field dressing and tumbler distribution patterns account for over 70% of the explained variance, while armatures, trampling, and defleshing make up the remaining 28% of explained variance. Overall, the full model explains 43% of the variance in edge damage, an increase of 11% over the single-variable model.

Points from Layers 6–9 (Fig 10E, primarily quartzite layers) are best fit by the full model consisting primarily of post-depositional trampling and tumbling edge damage distributions with defleshing and armatures explaining lesser amounts of the explained variation. The complete model explains 29% of the variation in Layer 6–9 point edge damage distribution compared to 14% by the single best-fitting parameter.

## Discussion

The KP1 points were previously argued to be best explained by a combination of spear-use and post-depositional processes [38] through assemblage edge damage distribution analysis of varying contributions and Kolmogorov-Smirnov hypothesis testing. The result here is mostly consistent with that prior result, however cutting processes may have also provided a significant amount of damage towards the resulting edge damage patterning than was previously noted. Trampling damage is more likely to occur when tools are at the surface where they are actively in contact with moving objects. Once artifacts become buried, their movement becomes more restricted and they are generally more protected by their surrounding matrix [67]. Since Stratum 4a at KP1 is located within a spring vent, it might be anticipated that damage patterning would be consistent with the tumbler distribution—which it is as a minor component of the best-fitting model. Wilkins et al. [15] excluded damage scars that had a surface color different from the rest of the patinated surface since those damage scars had to have occurred post-patination, and thus, post-behavioral input. When analyzed separately, the post-patination distribution on points from KP1 reported by Wilkins et al. is best-fit by trampling damage (R^2^ = 0.29). Therefore, trampling, or other natural processes that may mimic trampling, may have occurred once the artifacts had already been patinated. The R^2^ values for armatures at KP1 are much higher than at PP13B or DK1, which may be partly due to the post-patination (and thus, clearly taphonomic) damage that was able to be identified and analyzed separately [15]. The predominantly quartzite south coast assemblages did not exhibit patination, and thus was likely included as part of the overall edge damage distribution.

PP13B has a complex formational history, and the observation that the edge damage from both the MIS6 and MIS5 layers are most consistent with tumbler damage is further evidence of the compound influences of geologic and behavioral processes on the archaeological record. Micromorphology of the brecciated sediments associated with MIS6 suggests some degree of artifact transport due to induration and erosion of the sediments [120]. Although Schoville [39] found that disturbance intensity was not positively correlated with abundance of edge damage, the distribution of damage may provide evidence that a significant amount of taphonomic damage occurred. The fabric analysis performed by Bernatchez [121] illustrates that most artifacts at PP13B are subjected to disturbance intensity less than that from ‘shallow run-off’ processes, except for two stratigraphic aggregates occupied during MIS5 in the Western Area (LB Sand 1 and LBG Sand 2), which is also associated with the highest relative contribution of tumbling damage in the full best-fit model.

Points being used as cutting tools during MIS5 at PP13B was suggested by separate but similar analyses from Bird et al. [22] and Schoville [21]. The MIS6 and MIS5 points both suggest defleshing tasks which would be consistent with faunal transport to the cave location after field dressing. In both MIS6 and MIS5, approximately 30% of the explained variation in archaeological edge damage formation is consistent with defleshing experimental processes. Although only accounting for ~15% of the explained variance in edge damage for both MIS6 and MIS5, the evidence for quartzite points being occasionally used as armature-tips prior to discard is supported by the faunal evidence from PP13B. O’Driscoll [17] argues that three bones of size 3 mammals identified by Thompson [122] from PP13B have stone fragments embedded in them consistent with armature lesions. One of the fragments is from MIS6, and the other two are from MIS5. Although there is little evidence for spear-points based on the impact fractures from MIS5 [21], this could reflect patterns of broken tool discard on the landscape related to mobility and foraging strategies. Minimal input from armature use would be consistent with multifunctional tools deposited away from kill-sites.

The DK1 points provide two disparate samples when divided between Layers 10–14 and Layers 6–9. In the lower levels where silcrete is more abundant, points are most consistent with butchery processes that explains a fairly high amount of the overall variation in edge damage. The more specific model identifies field dressing as the most consistent experimental pattern. In Layers 6–9, post-depositional processes account for the majority of explained variance. Schoville [39] had suggested that edge damage on points from DK1 collectively is inconsistent with a taphonomic origin, but was unable to rule out damage being correlated with disturbance intensity because artifacts were not piece plotted. If both stratigraphic groupings are combined and analyzed with the experimental parameters tested here, the single-best fit is achieved from the field dressing distribution (R^2^ = 0.24). This suggests that the main behavioral signature from DK1 is butchery, and mainly from the lower Layers 10–14 since Layers 6–9 are most consistent with trampling damage. The geoarchaeological record at DK1 is consistent with trampling as well. Goldberg [105] noted that the lack of bedding visible in Layer 6 was consistent with trampling, while bone fragments in Layer 8 appeared rotated in micromorphology due to bioturbation or trampling processes. Intensive compaction, diagenesis, and roof-fall occurred in Layer 6, which is consistent with the trampling damage evidence on points from layers 6–9 [100]. Layer 12 also appeared to have bone displaced into unconsolidated sandy layers due to trampling, which may explain the contribution of trampling and tumbling edge damage patterning on points from Layers 10–14.

### Damage ‘Palimpsest’

McPherron et al. [116] identify edge angle as an important factor in the likelihood of edges to form damage due to taphonomic processes. Although others have shown that more acute angles form damage more readily [58, 71], McPherron et al. demonstrated that the distribution of edge angle is on average unequal around some flake classes. In other words, taphonomic edge damage may appear “patterned” in aggregate, or significantly different from a random distribution of damage, simply due to the patterned distribution of edge angle around tool perimeters. Often the distal portion of flake edges is thinner than near the platforms, so an increase in tip damage may be expected due to trampling. This has important implications for the initial studies that compared archaeological damage on points at PP13B to random, or uniform, distributions (e.g., [21, 22]). When archaeological patterns are significantly different from random, they may not be significantly different from the patterned damage signature due to post-depositional processes producing damage more frequently on the acute regions of detached lithic pieces.

Wilkins et al. [15] circumvented this issue by comparing point edge damage to post-patination taphonomic damage patterning on points. This is an important distinction, because a random distribution assumes damage will form with equal probability around the flake, which is unlikely to be true for points due to the patterned distribution of edge angle. However, the post-patination damage patterning on points includes the same patterned distribution of edge angle—there is no assumption of equal probability across the edge. The only assumption is an uniformitarian one—archaeological points that have edge damage due to trampling, will be statistically similar to experimental points that have damage due to trampling. McPherron et al. [116] state that, “edge angle needs to be controlled before it is possible to identify a signature that is indicative of a specific use-related pattern (p.79)”. However, when equivalent experimental and archaeological tool class edge damage distributions are compared (i.e., points to points, blades to blades, etc.), edge angle has been controlled for by the methodological design.

The approach applied here determines the known experimental processes that best fits the observed archaeological data. This study provides explanation for some of the variation in edge damage formation at PP13B, however over 80% is left unexplained. Increasing the number and variability of experimental processes that can be compared may explain more variability in archaeological edge damage formation than what was achieved by the experimental processes presented here, however this can be a starting point for such investigations. Tool handedness, hafting technique, hide scraping, and other behavioral processes would be useful distributions to compare archaeological data and start building a landscape-scale database of tool damage variability. By recording wear features within a GIS framework, these observations can be shared quickly and easily among researchers to test replicability and build a greater body of experimental wear patterning.

### MSA Behavioral Variability

Within the last 20 years, there has been a shift in how MSA behavioral adaptations are perceived [7, 9]. The traditional perspective viewed MSA foragers as less adept hunters, technologically less sophisticated, and culturally less complex than LSA and Upper Paleolithic hominins (e.g., [123]). Now it is widely recognized that MSA hunters were highly skilled at acquiring diverse and ‘dangerous’ taxa and scavenging was not their predominant method of acquiring meat [18, 26, 124]. Tortoises, shellfish, and mole-rats were frequently obtained in parallel with the acquisition of large prey [29, 95, 125]. MSA technology includes many novel techniques for constructing tools, including heat-treatment of silcrete, pressure-flaking, and the use of complicated ochre mastic recipes for hafting [126–129]. Artifacts with symbolic purposes have been found from several MSA sites, including shell beads, cross-hatched ochre incisions, ‘beauty’ shells, and engraved ostrich eggshell [130–133]. This study fits within this paradigm shift by exploring the emergent complexity seen in the manufacture, use, and discard of stone tools in the MSA. This approach allows variability in technological behaviors to be explored at multiple scales that provide insight into early modern human behavior. There is no single point “function”, rather varying contributions of different behavioral processes in some situations were overlain with postdepositional damage. As previously noted, some studies have emphasizes MSA points for cutting, and others have emphasized their use as spear-points. These distinctions may be a result of how foragers structured their landscape in response to resource extraction behaviors in the MSA. Lithic tools break frequently during use and the stone often discarded. Where and when certain sites were occupied by hunting parties, residential camps, logistical forays, or long-term settlements has implications for how and when tools are being used, and subsequently broken and discarded. Points at KP1 were likely used as armature tips prior to being discarded, whereas the occupations at PP13B and at DK1 in Layers 10–14 may have tended to use points for cutting activities prior to discard. However, the low R^2^ values at PP13B suggest additional processes of edge damage formation that were not identified in this study may be more consistent with the archaeological patterning than those examined here. Although the sample of assemblages is small, the association of cave assemblages with primarily cutting tools and the open-air site with armature tips may reflect a general pattern in how tools were used and discarded in the MSA. MSA foragers adjusted their technological system based on needs on the landscape, and this is likely reflected in the variability in edge damage observed on points.

## Conclusion

The study presented here provides edge damage data on four general experimental processes—spear tipped armatures, butchery, trampling, and tumbling. These distributions are then incorporated into stepwise regression models for MSA archaeological assemblages from KP1, PP13B, and DK1. This analysis provides support for the interpretation of points from KP1 as spear tipped armatures. Points from PP13B and DK1 are more variable, with taphonomic, post-depositional damage being the single best-fitting variable at PP13B as well as layers 6–9 at DK1. It is perhaps relevant that these layers are most associated with coastal occupation (i.e., shellfish is present). Coastal occupation may have been more intensive than when the caves were further inland away from coastal resources during periods of lowered sea-level [134], and tools may have been exposed to greater amounts of taphonomic processes during these times.

An advantage of the model-fitting approach is that multiple processes can be identified, which allows a palimpsest of edge damage formation processes to be elucidated from archaeological data. At PP13B, in addition to taphonomic patterning, MIS5 layers are consistent with contribution of damage from defleshing butchery processes, as well as tumbling damage, and potentially use as armatures and field processing. Similar processes are inferred from the MIS6 points. At DK1, layers 6–9 are most consistent with post-depositional damage formation, whereas earlier layers 10–14 are most consistent with field dressing processes in addition to tumbling, and minor contribution from armature use and trampling edge damage processes.

This study provides the experimental basis and an analytical methodology with which multiple edge damage processes from archaeological assemblages can be identified. Incorporating these into a landscape scale framework can provide insight into how MSA technologies were constructed, used, maintained, and discarded. Although damage may at first appear to be distributed as a ‘blur’, the edge damage distribution method can provide new insights into MSA technological behavior and site formation histories.

## Supporting Information

S1 File Archaeological specimen numbers. List of archaeological points with observed edge damage, raw material, and corresponding museum repository.
(XLSX)

S2 File Point Metrics and Damage. Individual experimental and archaeological point metrics and edge damage occurrences along edge perimeter. Each edge defined as segment of tool perimeter between platform (0) and most distal point away from the platform (i.e., tip at 100). Edges are classified based on how they appear in photograph with platform down, so that the dorsal left edge corresponds to the underside of the ventral right when the tool is turned over (and dorsal right corresponds to other side of ventral left).
(XLSX)

S3 File Distribution data with loess smoothed curves. Aggregated edge damage distribution data from experimental and archaeological point assemblages.
(XLSX)
